# COVID-19 Identification from Low-Quality Computed Tomography Using a Modified Enhanced Super-Resolution Generative Adversarial Network Plus and Siamese Capsule Network

**DOI:** 10.3390/healthcare10020403

**Published:** 2022-02-21

**Authors:** Grace Ugochi Nneji, Jianhua Deng, Happy Nkanta Monday, Md Altab Hossin, Sandra Obiora, Saifun Nahar, Jingye Cai

**Affiliations:** 1 School of Information and Software Engineering, University of Electronic Science and Technology of China, Chengdu 611731, China; ugochinneji@std.uestc.edu.cn (G.U.N.); jianhua.deng@uestc.edu.cn (J.D.); 2 School of Computer Science and Engineering, University of Electronic Science and Technology of China, Chengdu 611731, China; mh.nkanta@std.uestc.edu.cn; 3 School of Management and Economics, University of Electronic Science and Technology of China, Chengdu 611731, China; altabbd@uestc.edu.cn (M.A.H.); sandra_ora2000@hotmail.com (S.O.); 4 Department of Information System and Technology, University of Missouri St. Louis, St. Louis 63121, MO, USA; snnnm@umsl.edu

**Keywords:** computed tomography, super-resolution, deep learning, adversarial learning, Siamese network, convolutional neural network

## Abstract

Computed Tomography has become a vital screening method for the detection of coronavirus 2019 (COVID-19). With the high mortality rate and overload for domain experts, radiologists, and clinicians, there is a need for the application of a computerized diagnostic technique. To this effect, we have taken into consideration improving the performance of COVID-19 identification by tackling the issue of low quality and resolution of computed tomography images by introducing our method. We have reported about a technique named the modified enhanced super resolution generative adversarial network for a better high resolution of computed tomography images. Furthermore, in contrast to the fashion of increasing network depth and complexity to beef up imaging performance, we incorporated a Siamese capsule network that extracts distinct features for COVID-19 identification.The qualitative and quantitative results establish that the proposed model is effective, accurate, and robust for COVID-19 screening. We demonstrate the proposed model for COVID-19 identification on a publicly available dataset COVID-CT, which contains 349 COVID-19 and 463 non-COVID-19 computed tomography images. The proposed method achieves an accuracy of 97.92%, sensitivity of 98.85%, specificity of 97.21%, AUC of 98.03%, precision of 98.44%, and F1 score of 97.52%. Our approach obtained state-of-the-art performance, according to experimental results, which is helpful for COVID-19 screening. This new conceptual framework is proposed to play an influential task in the issue facing COVID-19 and related ailments, with the availability of few datasets.

## 1. Introduction

Coronavirus disease 2019 (COVID-19) has become a pulmonary ailment instigated by severe pneumonia diseases. Wuhan city of Hubei province in China is the ground-zero of the epidemic where COVID-19 was first discovered in December 2019, and has since escalated around the world, resulting in the ongoing coronavirus pandemic of 2021. As of 3 January 2022, there has been over 290 million confirmed cases and over 5.4 million deaths globally [1,2]. The reverse transcription polymerase chain reaction, RT-PCR, being an accepted procedure for diagnosing COVID-19, is manually done to perform a viral nucleic acid test by using nasopharyngeal and throat swabs seen to be affected by sampling errors and low viral load. Additionally, RT-PCR [3] is complex and time consuming, and it requires multiple tests for a definitive result and relatively low sensitivity. There are insufficient test-kits and domain professionals in the clinics, and a swift increase in the value of infected patients demand for an automatic screening application, which serves as an alternative method for medical professionals to hastily identify the infected patients who need instant isolation and additional clinical verification. Alternative screening methods [4,5,6] have been established for the COVID-19 identification, which employs chest X-ray or computed tomography [7].

Several studies have presented different methodologies using artificial intelligence to diagnose COVID-19. Some utilized CXR images while others applied CT images. In this section, we present relevant studies on both CXR and CT images sequentially. Given enough data, CNNs have achieved many accomplishments in a diverse area of medical diagnostic imaging [8]. This degree of efficiency is achieved by training on labeled data and fine-tuning the system’s millions of parameters. Deep learning systems are being used in a variety of published research for COVID-19 diagnosis and screening. The ImageNet weights were pre-trained on a design 18-layer residual network against 100 COVID-19 and 1431 Pneumonia X-ray datasets [9]. COVIDX-Net is a collection of Deep Learning frameworks that were trained on a limited 25 verified COVID-19 CXR dataset [10]. COVID-19, healthy, and viral pneumonia CXR images are included in a tradition curated dataset [11]. In addition, a traditional residual CNN which better differentiates COVID-19 CXR from healthy and other Pneumonia CXR images was reported in [12]. The X-ray samples which contain the COVID-19, normal, and pneumonia scans were collected from the RSNA dataset [13].

Recent studies have focused on an automatic diagnostic procedure of COVID-19 pneumonia from CT scans, with positive results [14,15,16,17] of high accuracies using a non-public dataset of CT images. A report of a hybridized 3D classification CNN integrated U-Net segmentation algorithm was utilized to effectively detect the existence of COVID-19 CT in [18].

Another paper proposed the COVID-Net, which is a CNN-based model for the detection of COVID-19 cases using CXR images. Using the COVIDx dataset, an accuracy of 93.3% and sensitivity of 91.0% were actualized. A Pre-trained ResNet50 CNN with ImageNet weight adequately identified COVID-19 with 94% accuracy using private CT datasets in [19] compared to a CT slice in normal conditions. The use of machine learning discriminating between community-acquired pneumonia and COVID-19 CT scans was tackled instantly [20]. For pulmonary field segmentation, this device uses a U-Net pre-processor model; after that, a 3D ResNet50 architecture with ImageNet weights was transferred. According to the authors in [21], they proposed using three different channels of CXR images with their individual deep neural networks. Thus, the final feature weights of the three channels are concatenated and softmax classification is utilized to determine the final classification of COVID-19 and other pneumonia. Different feature extraction approaches on patches were reported using SVM as a classifier. This realized the best classification value of 99.68% using 10-fold cross-validation and the GLSZM feature extraction method in stage 2 using CT scans according to authors in [22].

A segmented CT scan in [23] was proposed to get rid of the disease and the pulmonary fields, then images were categorized based on infection size using an infection size conscious-based random forest classifier approach. Using 5-fold cross validation, the procedure achieved an average 94% AUC on 1027 healthy and 1658 COVID-19 instances. In another case study, authors in [24] built and deployed an AI system which automatically analyzes CT images to detect COVID-19 pneumonia characteristics. Using training samples of 1136, accuracies of 95.5%, sensitivity of 97.4%, and specificity of 92.2% were achieved. Another work [25] proposed the use of an AI system for the detection of COVID-19 from other pneumonia cases and on the testing set of 3199 CT scans; an AUC for the multi-class achieved 97.81%. The authors in [26] collected CT scans of patients from two hospitals in China to detect COVID-19 and other pneumonia cases. On the binary class, an accuracy of 86%, recall of 96%, and specificity of 77% were achieved, whereas, for the multi-class, the accuracy was reported as 93%, sensitivity as 93%, and that of specificity as 93%. Detecting quantitative distinct features of CT scans, the authors in [27] assessed the severity of COVID-19 and achieved an accuracy of 87.5%, true positive rate (sensitivity) of 93.3%, and true negative rate (specificity) of 74.5% using a machine learning approach.

Deep Learning architecture was suggested in [28] to adequately segment infectious areas. Patches of infected regions were incorporated in a ResNet-18 algorithm for the classification of three labels, namely, healthy, COVID-19, and Influenza-A patients with distances from the edge of the lung reaching an accuracy of 86.7%. Using an in-house dataset, Wang et al. [29] used an inception network to diagnose COVID-19 from CT images. They registered an internal validation set with a cumulative accuracy of 89.5%, 88% specificity, and 87% sensitivity while the external validation set records a cumulative accuracy of 79.3%, 83% specificity, and 67% sensitivity. The external validation collection consists of 100 samples each for healthy and pneumonia instances, whereas COVID-19 has just 10 instances. Because of the large number of trainable parameters, it is easy for CNNs to overfit on a small amount of instances. As a result, generalization efficiency is relatively equivalent to the proportion of the data class. Tiny datasets are the most difficult task in the medical imaging domain because of the restricted quantity and variety of samples.

Singh et al. [30] proposed an ensembling method for automatically screening COVID-19 screening utilizing CT scans. The suggested ensemble model used three well-known models, DCCNs, ResNet152V2, and VGG16, to solve the sensitivity issue associated with RT-PCR, thereby obtaining a sensitivity of 98.8%. Scarpiniti et al. [31] suggested a method of Deep Denoising Convolutional Autoencoder to screen COVID-19 using CT scans by taking advantage of the compact and hidden representation of the model. This model utilized an unsupervised scheme trained on CT exams to create a statistical representation for obtaining a target histogram. The method achieved a maximum accuracy of 100% and comparably high values for other metrics. Khan et al. [32] proposed a contrast enhancement scheme by combining a top-hat and Wiener filter using parallel fusion and optimization of pre-trained deep learning frameworks of VGG16 and AlexNet to automatically extract and fuse features for COVID-19 screening using CT scans to obtain 98% accuracy. Rehman et al. [33] presented a two-way classification technique using chest X-ray modality to diagnose 15 different forms of chest disorders, including the COVID-19 condition and achieved 99.98% accuracy.

Adversarial learning [34], a technique that allows CNNs to acquire feature mappings from intricate information dispersion with remarkable accuracy has recently gained popularity. GAN is a mini-max game for which the generator G and the discriminator D are opposite players. In this game, G is taught how to map source images x in a target domain X to reference images y in a source domain Y. D, on the other hand, uses a binary label to differentiate between the produced and target images y. Once properly trained, GAN may design a high-dimensional representation of image features. Medical image collection is a time-consuming and costly procedure that necessitates the involvement of radiologists and researchers [35]. The computed tomography is amongst the most famous medical imaging techniques for monitoring, diagnostic, and image-guided intervention [36]. High-resolution CT imaging has the potential to improve the quality of radiomic features as well. As a result, in the CT region, super-resolution (SR) methods are getting a lot of attention [34]. The size of the CT focal spot, the recapture algorithms, the detector element pitch, and other considerations all limit the image resolution of a CT imaging device. Despite the fact that modern CT imaging and visualization tools can produce any little voxel, the innate resolution is still far below what is needed in critical applications. As a result, processing HR CT images with the lowest possible radiation portion is highly desirable in the CT region. Low resolution (LR) and poor quality images remain a major challenge in AI-based COVID-19 diagnosis systems. As a result, the AI-based system would learn inconsistency and noise from the data, losing out on the distinguishing characteristics which would have been retrieved for optimal diagnosis. We suggested modified enhanced super-resolution GAN for COVID-19 identification to resolve these issues.

This work’s contribution is as follows: (1) To the best of our knowledge, this is the first study to combine modified enhanced super-resolution GAN with a Siamese capsule network for COVID-19 diagnosis. The modified enhanced super-resolution generative adversarial network used in this study generates super-resolution CT images (SR) from low-resolution CT images of which distinct information can be extracted. The MESRGAN addresses the problem of low quality images and helps to remove noisy artifacts generated by GAN due to the decompressing nature of JPG format images. (2) To reduce the computational overheads and avoid framework, we incorporated a Siamese capsule network. The super-resolution CT images achieved from the MESRGAN+ are directly sent to the Siamese capsule network for identification of COVID-19 in an end to end fashion, resulting in improved screening accuracy.

The following is how the rest of the paper is organized: The approach is explained in detail in Section 2. Section 3 contains a summary of the dataset, implementation information, and experimental results. We conducted robustness validation and result in Section 4 and presented the findings as well as other relevant discussion in Section 5. In Section 6, we present the conclusions of this study.

## 2. Materials and Methods

This section presents the problem statement, dataset, and preprocessing procedure used in this study. Next, we explained the Siamese capsule network and modified enhanced super-resolution GAN plus based on low quality images for COVID-19 identification (MESRGAN+ Siamese-CapsNet). Lastly, we provided the implementation details for our proposed model.

### 2.1. Problem Statement

Effective COVID-19 screening is needed in light of the looming pandemic threat. The lack of COVID-19 test kits in many developed or rural areas, as well as the time taken to produce the samples (proper) results, poses a significant problem for developing countries with under-equipped hospitals and clinics. Developing countries frequently lack sufficient COVID-19 kits, limiting primary healthcare clinics’ ability to receive, ship, and analyze, forcing them to rely more on specialized centers in providing them with the test results. To react to the third wave of the pandemic in areas with low access to viral or antibody tests, which can be useful in COVID-19, plays a major role, an automated and efficient method is required to meet the increasing demand for new test cases. Many studies have shown that CT scans can detect ground–glass opacities and other chest features that are higher in resolution than those of a normal CXR [37]. Nevertheless, CT might seem to be comparatively costly for installation and maintenance but yet its scans are reliable for the automatic detection of COVID-19. However, in AI-based CT detection systems for large-scale imaging, there are two major bottlenecks:

(1) The low resolution (LR) features is a problem.

(2) The collected dataset samples are often limited and may include fuzzy and meaningless data.

Additionally, experienced radiologists have difficulty distinguishing between the symptoms of COVID-19 pneumonia and community-acquired bacterial pneumonia while examining CT images [38]. Furthermore, the influx of patients into hospital ERs during the pandemic, manual analysis of radiograph data, and accurate decision-making would all lead to a difficult trade-off between accuracy and detection time, potentially exhausting the radiologist unit, hence necessitating the use of an automated identification process. Third-wave COVID-19 activity would necessitate an increase in an automatic system to help contain the further spread of the virus by proposing a deep learning resolution-based GAN and identification network.

### 2.2. Datasets

In this study, we used an open-source dataset called a COVID-CT dataset. The dataset was obtained from Yang et al. [39] and consists of 349 COVID-19 CT images from 216 patients and 463 non-COVID-19 CTs. These CT scans have different sizes, whereby the minimum to maximum height is between 153 and 1853; the minimum to maximum width is between 124 and 1485.

### 2.3. The Proposed Framework: Modified Enhanced Super-Resolution Generative Adversarial Network with a Siamese Capsule Network

In this study, our proposed model is subdivided into three stages. Firstly, the CT images of arbitrary resolution are passed to the image scaling-adaptive module for resize. Secondly, the super-resolution architecture, called the Modified Enhanced Super Resolution GAN (MESRGAN+), is used to recreate LR images into high-resolution and eliminate compression artifacts. Finally, the reconstructed HR image is passed to the Siamese capsule network to extract and learn discriminative features for the screening of COVID-19.

#### 2.3.1. Image Scale-Based Adaptive Module

**Figure 1:**
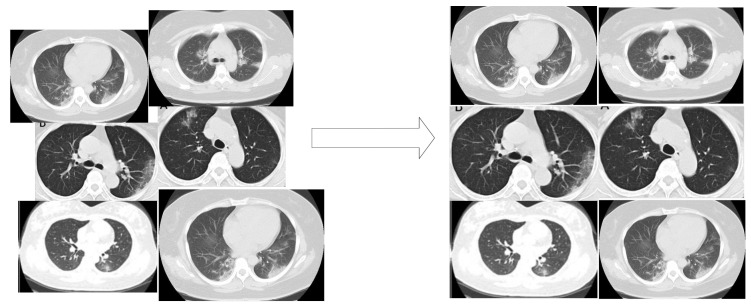
Scaling images at different resolutions to a fixed resolution using an image scaling adaptive module.

During the training phase, the OpenCV image scaling method adjusted the different resolutions to a fixed resolution of 224×224 as width and height, respectively, as seen in Figure 1 before passing through the MESRGAN+ framework.

#### 2.3.2. Modified Enhanced Super Resolution GAN Plus (MESRGAN+)

In this study, our aim is to enhance the low quality CT images into a super-resolution before passing through the Siamese capsule network for COVID-19 identification. We will present the proposed modified enhanced super resolution generative adversarial network plus (MESRGAN+) architecture and its structural improvement for achieving a balance in perceptual quality and PSNR in this section. Hence, we will briefly highlight the transition of SRGAN to MESRGAN+.

##### Transition of Super Resolution by GAN

**Figure 2:**
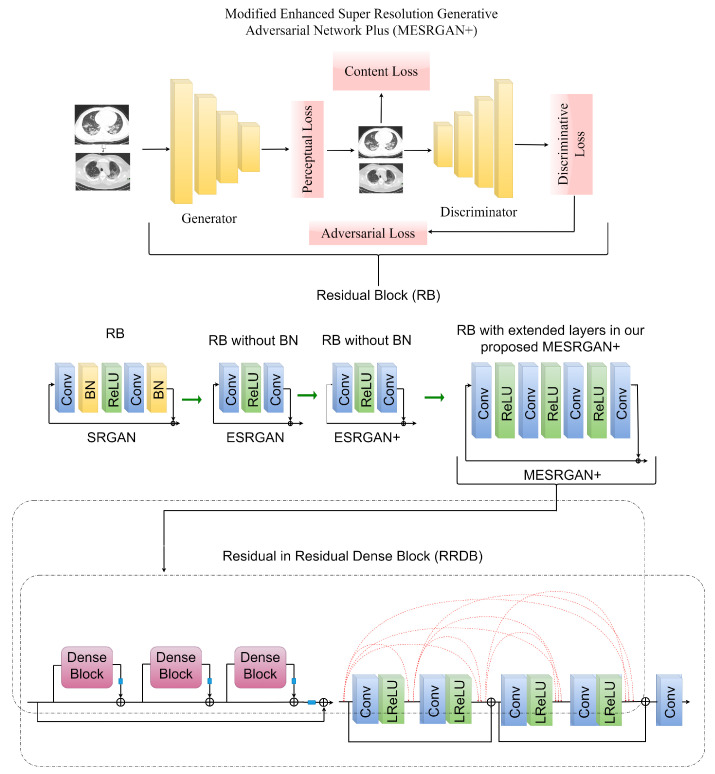
A structural configuration of ESRGAN+ where feature extraction and most computation are performed on the LR image feature. We re-design the structure for better optimization and performance by making a few modifications to the generator structure. The transition from SRGAN to MESRGAN+ is equally showcased.

SRGAN [40] utilizes basic blocks of a deep residual network to recover image-realistic details in which batch normalization (BN) is followed after each convolutional layer as depicted in Figure 2. The transition from SRGAN to ESRGAN [41] is based on two modifications; the first modification is the removal of all BN in the generator structure and the second modification involves the replacement of the original basic block with a Residual-in-Residual Dense Block (RRDB) as shown in Figure 2. Finally, the transition from ESRGAN [41] to ESRGAN+ [42] is based on introducing an additional level of residual learning every two layers inside the dense block as illustrated in Figure 2 without changing the convolutional structure.

##### MESRGAN+ Architecture

In our proposed super resolution architecture, the overall structural configuration of the Residual-in-Residual Dense Block (RRDB) in ESRGAN+ is kept the same as shown in Figure 2. We made few modifications to the ESRGAN+ network in the generator structure by expanding the convolutional layers with two additional convolutional layers and two ReLU activation functions. Normally, the direct mapping of the high-dimensional LR features to HR feature vectors ultimately results in high computational complexity, and we know that the dimension of the LR feature is normally very huge. To address this bottleneck, we utilize a 1 × 1 convolutional layer as the second layer to reduce the computational cost by shrinking the LR dimensional features, thereby maintaining the same kernel size of 64 after the first layer. In order to maintain consistency and the performance of ESRGAN+, we utilized a 3 × 3 filter size and a kernel size of 64 for the third and fourth convolutional layers.

To produce the high-resolution images from the scale-adaptive module, the scale factor is increased to 4. This image’s network generator produces $v^{k+1}=G_{k}\left(v^{k}\right)$. The feature map is extracted to calculate the perceptual loss before being passed to the final activation function. Pixel-wise loss is measured, and the created image is forwarded to the discriminator network to differentiate between the created image $v^{k+1}$ and the actual image ${\hat{v}}^{k+1}$. This actual image ${\hat{v}}^{k+1}$ is fed to the discriminator network for training, which results in the same super-resolution image $v^{k+1}$. Then, the generator network recalculates the loss function and produces the same image. This entire process was only completed when the discriminator network could no longer tell the difference between real and fabricated images. We train the generator function $G_{k}$ to approximate the HR of the next LR image ${\hat{v}}^{k+1}$, which LR input can represent. In Equation (1), the total super-resolution network is calculated as:

(1) $$\Pi_{Totalloss}=\Pi_{Gen}\left(\Pi_{Perceptualloss}+\mu \Pi_{G}^{Ra}+\eta L1\right)+\Pi_{Dis}^{Ra}$$

As Equation (1) is evaluated, the $\Pi_{G}^{Ra}$, known as the adversarial loss, is the loss of a relativistic generator, the content loss is L1, μ, and η are coefficients to offset the losses. $\Pi_{Gen}$ is the generator loss, $\Pi_{Perceptualloss}$ is the perceptual loss, and $\Pi_{Dis}^{Ra}$ is the discriminator loss.

##### Perceptual Loss

Perceptual loss works to improve the texture and picture accuracy of the generated images [43]. Euclidean distance is used to compare the feature maps of the original image ${\hat{v}}^{k+1}$ and the generated image $v^{k+1}$. According to the definition of [43], the feature map was extracted before using the generator network’s final activation function. In the COVID-19 identification, the illumination difference occurs in the CT image datasets obtained from the source. The extraction of feature maps after activation function caused the model to have inconsistent illumination, directly impacting the model output. When recapturing HR from LR, it provides close supervision between feature maps. The fact that CT images are not sufficiently HR is well understood, and this aspect boosts model re-generation dramatically. Mapping feature $\alpha_{ij}$ is obtained after *j*th-convolution and before the max-pooling layer. The formality is measured as the distance between the function representations of the super-resolution image $G_{k}\left(v^{k}\right)$ and the real image ${\hat{v}}^{k+1}$. Formal calculation between feature maps is given in an algebraic Equation (2):

(2) $$\Pi_{Perceptualloss}=\sum_{x=1}^{W_{ij}}\sum_{y=1}^{H_{ij}}{\left(\alpha_{ij}{\left({\hat{v}}^{k+1}\right)}_{xy}-\alpha_{ij}{\left(G_{k}\left(v^{k}\right)\right)}_{xy}\right)}^{2}$$

Instead of penalizing the output image $v^{k+1}$ precisely the same as the input image ν${\hat{v}}^{k+1}$, perceptual loss prefers the representation to be identical.

##### Content Loss

By manipulating the HR image $v^{k+1}$ to be close to the ground truth *v*${\hat{v}}^{k+1}$, the network improves the accuracy of pixel-level by calculating the L1-norm distance between both the ground truth and the recovered image. Compared to the L2 loss, which often results in over smooth results, the L1 loss is used for better efficiency and convergence. Equation (3) calculates the L1-norm distance between the SR image ${\left(G_{k}\left(v^{k}\right)\right)}_{xy}$ and the ground truth ${\left({\hat{v}}^{k+1}\right)}_{xy}$ given as:

(3) $$Ł1=\sum_{x}^{W}\sum_{y}^{H}{G_{k}{\left(v^{k}\right)}_{xy}-{\left({\hat{v}}^{k+1}\right)}_{xy})}_{1}$$

##### Relativistic Loss

The majority of the preliminary research focused on standard GAN. Meanwhile, we employ a rational discriminative loss in our SR network, ensuring that HR photos are not stylized or unrealistic. In Equations (4) and (5), the classification of the images is using the standard discriminator $D_{is}$ in GAN:

(4) $$D_{is}=\sigma \left(f_{d}\left({\hat{v}}^{k+1}\right)\right)\to 1$$

(5) $$D_{is}=\sigma \left(f_{d}\left(v^{k}\right)\right)\to 0$$

Equations (4) and (5) reflect the regular GAN’s operation. $D_{is}$ is the discriminator’s output to classify whether the images are real or artificial. The vector feature discriminator is represented as $f_{d}\left(.\right)$. Additionally, the word “σ” stands for the sigmoid function. Adversarial loss uses a binary classifier to make sure that what you obtain is true or not. We use the relativistic GAN [42] to distinguish between the real ${\hat{v}}^{k+1}$ and created data $G_{k}\left(v^{k}\right)$ with the distance computed as:

(6) $$D_{Ra}\left({\hat{v}}^{k+1},G_{k}\left(v^{k}\right)\right)$$

RGAN produces images with sharp edges when used in a relativistic model and provides more graphic and detail information than a typical GAN. It is seen in Equations (6) and (7) that RGAN is presented as:

(7) $$D_{Ra}\left(Real,Fake\right)=C\left(Real\right)-E\left(C(Fake)\right)\to 1$$

Equation (7) analyzes how realistic an image is compared to a fake one:

(8) $$D_{Ra}\left(Fake,Real\right)=C\left(Fake\right)-E\left(C(Real)\right)\to 0$$

Equation (8) analyzes how realistic an image is compared to a fake one. Here, E(.) is the average of all real or fake data in the sample. This slight modification makes the model more efficient than the standard discriminator network. The discriminator network loss is given in Equation (9) defined below:

(9) $$\Pi_{Dis}^{Ra}=-E_{{\hat{v}}^{k+1}}\left[log\left(D_{Ra}\left({\hat{v}}^{k+1},G_{k}\left(v^{k}\right)\right)\right)\right]-E_{G_{k}\left(v^{k}\right)}\left[log\left(1-D_{Ra}(G_{k}\left(v^{k}\right),\left({\hat{v}}^{k+1}\right)\right)\right]$$

Despite this, Equation (10) illustrates the adversarial loss for the RGAN:

(10) $$\Pi_{G}^{Ra}=-E_{{\hat{v}}^{k+1}}\left[log\left(1-D_{Ra}\left({\hat{v}}^{k+1},G_{k}\left(v^{k}\right)\right)\right)\right]-E_{G_{k}\left(v^{k}\right)}\left[log\left(D_{Ra}(G_{k}\left(v^{k}\right),\left({\hat{v}}^{k+1}\right)\right)\right]$$

The network is concurrently trained for both actual image ${\hat{v}}^{k+1}$ and created image $G_{k}\left(v^{k}\right)$ to minimize the failure of the discriminator and generator networks. When the discriminator gradient hits its optimum point $(1-D_{{\hat{v}}^{k+1}})\to 0$, i.e., discriminates between authentic images, it stops learning actual content ${\hat{v}}^{k+1}$ and focuses on generated images $G_{k}\left(v^{k}\right)$. At this level, custom GAN does not learn how to create more realistic images. In comparison, RGAN studies both images and gradients are dependent on both of terms, i.e., ${\hat{v}}^{k+1}$ and $G_{k}\left(v^{k}\right)$.

### 2.4. Siamese Capsule Network for COVID-19 Identification

**Figure 3:**
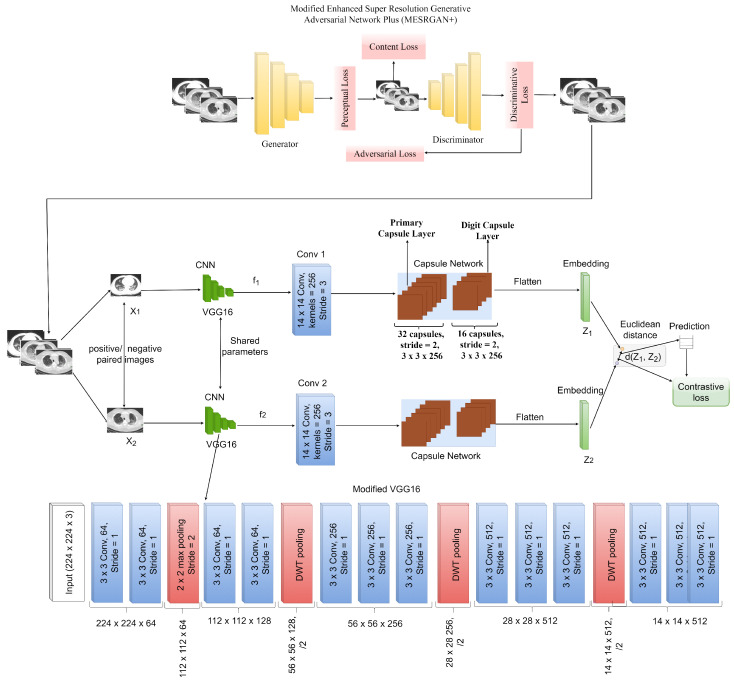
Our proposed modified Enhanced Super-Resolution Generative Adversarial Network Plus (MESRGAN+) and Siamese Capsule Network (Siamese-CapsNet).

Traditional CNN has achieved tremendous results in feature extraction of target images. Furthermore, the primary aim of the pooling layer is to achieve dimensionality reduction, but it could also lead to loss in spatial details, large rotation, and directional movement of the target images. This is one of the reasons for CNN’s unsatisfactory classification performance. To this effect, we proposed a Siamese capsule network sharing the same weights and parameters to effectively identify COVID-19. For classification problems, the authors in [44] were the first to propose a capsule network. A capsule network is made up of entity-oriented vectorial capsules, unlike traditional CNNs that employ scalar neurons to represent the probabilities of the presence of specific features. A capsule can be thought of as a vectorial grouping of neurons [44]. A capsule’s initialization parameters reflect a certain type of entity, and the capsule’s length denotes the likelihood of that entity’s occurrence. In retrieving intrinsic and distinguishing properties of entities, capsule networks are far stronger and more robust than ordinary CNNs. They have been shown to outperform humans in a variety of classification tests [44,45,46]. As a result, we modify the existing capsule network to create a multi-layer deep convolutional capsule network to achieve promising COVID-19 identification performance. A detailed illustration of our proposed architecture of a Siamese capsule Network with a CNN VGG16 pre-trained model as the backbone network is presented in Figure 3.

## 3. The Proposed MESRGAN+ Siamese Capsule Network

The proposed end-to-end framework for COVID-19 identification consists of a modified enhanced super resolution generative adversarial network (MESRGAN+) and Siamese convolutional capsule network (CapsNet) as seen in Figure 3. The MESRGAN+ model functions as the image quality enhancement network that generates high-resolution CT images from low-resolution counterparts. The MESRGAN+ model generates a high-resolution output image of size 896×896 also known as the reconstructed high-resolution image from the low-resolution input image of size 224×224. The high-resolution output of the MESRGAN+ becomes the new input image that is fed to the Siamese capsule network for feature extraction and identification of COVID-19. At this stage, the input size of the reconstructed high-resolution CT images is resized to 224×224×3 after the super-resolution operation without image quality distortion. In this paper, we utilized a VGG16 model as the feature extractor due to its performance on an image classification task. VGG16 has 13 convolutional layers arranged in blocks, three fully connected layers, and five max pooling layers. Due to the discriminative features of the CT dataset, the first few convolutional layers captures low-level features which include curves, color, edges, and texture, whereas the high-level features are captured as the convolutional layers get deeper. To achieve better identification performance and maintain the integrity of high-level features, it is necessary to extract the high-level features of the CT images by removing the max-pooling layers in the second, third, fourth, and fifth blocks and replace it with discrete wavelet pooling layers in order to reduce the loss of spatial details to the minimum in order to achieve reduction in dimension without losing positional details. More to the point, the choice of VGG16 network as the backbone model is because of the reasonable network depth without performance degradation as compared to other deeper convolutional networks. In our proposed model, the spatial details are transformed into Primary Capsule layers (PrimaryCaps) in the form of capsules after the feature extraction stage. Routing by agreement is introduced to learn the spatial details in the form of a transformation matrix. The connection strength of the digit capsule is controlled by the routing coefficient. The probability that the capsule instance exists is represented by the length of the feature vector, which is compressed to 1 with the help of a nonlinear function. We added a convolutional layer to each branch of the network in order to reduce the dimension. We introduce a regularization term to enhance the robustness of the model and, finally, the similarity score is calculated using Euclidean distance as a distance matrix term to verify whether the images belong to the same label or not. Since the output is represented by the length of the feature vector, which is the probability that the capsule instance exists, it is sent to the classifier unit for prediction of its corresponding label. A key characteristic of this capsule formulation, which is impossible to achieve with CNN scalar neuron models, is that the vectorial representation permits a capsule to learn and detects variants of a feature. To capsule *j*, Cj, the overall input in the convolutional capsule layers is the weighted summation of all predictions obtained from the capsules inside the convolutional kernel as seen in Equation (11):

(11) $$C_{j}=\sum_{i}a_{ij}\cdot U_{j/i}$$

where $C_{j}$ represents the sum total input to capsule *j*; the indicator that depicts the degree of which capsule *i* activates capsule *j* is called the coupling coefficient $a_{ij}$. The prediction $U_{j/i}$ from capsule *i* to capsule *j* is illustrated below in Equation (12):

(12) $$U_{j/i}=W_{ij}\cdot U_{i}$$

$U_{i}$ denotes the capsule i’s output and $W_{ij}$ represents the weight network on the edge linking capsules *i* and *j*. A robust routing method [44] decides the coefficient between capsule *i* and other linked capsules in the above layer summing to 1. To ignite another capsule, the robust routing method, known as routing by agreement, considers both the length of a capsule and its instantiation parameters. This differs from traditional CNN models, which simply evaluate probability. As a result, capsule networks are reliable and more powerful at abstracting inherent object characteristics. Recall that the capsule’s length is used to determine the likelihood of an entity’s presence. A nonlinear “squashing” function is used as the activation function for the convolutional capsule layers in ensuring a perfect probability estimation where capsules with short vectors have low probability— otherwise, high probability all at maintaining a constant orientation. Squashing function is given in Equation (13) as:

(13) $$U_{j}=\frac{\;||C_{j}{\left|\right|}^{2}}{1+\left|\right|C_{j}{\left|\right|}^{2}}\cdot \;\frac{C_{j}}{\left|\right|C_{j}\left|\right|}$$

The max-pooling layer in the capsule performs feature down-sampling in order to reduce the network size. However, we adopted DWT pooling of $K_{m}$×$K_{m}$ to replace max-pooling layer and stride of $K_{m}$ direct to the feature maps of the last convolutional capsule layer. The only capsule with the longest vector is kept while the rest are discarded in the $K_{m}$×$K_{m}$ kernel. To this effect, the size of the network and capsule amount are reduced and leave a selection of the essential capsules. A high-level entity abstraction with a global view is flattened to arrive at the feature embeddings. In addition, dynamic routing between two connected capsule layers is utilized, and the squashing function is used to normalize capsule outputs.

In this study, we adopted a VGG16 pre-trained network as the backbone feature extractor in our proposed Siamese capsule network. We fine-tuned the network by replacing the max-pooling layers in each convolutional block with discrete wavelet transform (DWT) pooling except for the first convolutional block, which retains its max-pooling layer. Furthermore, the max-pooling layer in the last convolutional block is completely eliminated without a replacement in order to arrive at a feature tensor of 14×14×512 as the final output from the input image. Before passing the extracted features to the primary capsule (PrimaryCaps), a convolutional layer of 256 kernels size and 14×14 filter size is introduced to reduce the dimension of the image feature which is then used as the input to the PrimaryCaps.

We added a dropout of 0.5 before the PrimaryCaps to avoid over-fitting. The PrimaryCaps consists of a 6D convolutional capsule layer of 32 channels and each PrimaryCaps consists of six convolutional units. In total, the PrimaryCaps has a dimension of 14×14×32 capsule outputs, where each output is a 6D vector. The DigitCaps consists of 12 convolutional capsules per class and each capsule is a 12D vector which is connected to all capsules in the PrimaryCaps. In total, DigitCaps has an M number of capsule outputs, where M corresponds to the number of classes in the dataset. Furthermore, another dropout of 0.5 was introduced to avoid over-fitting during the transmission of the two capsule layers. The feature tensor is reduced to 7×7×1024 by introducing DWT pooling. Finally, with the use of the L2-normalization layer, the distance matrix between the feature tensors is computed and followed by a fully connected layer for classification. During training of our Siamese CapsNet model, we trained our model for 40 epochs and a batch size of 16 with an Adam optimizer and learning rate of 0.0002. Furthermore, the proposed method has been evaluated on the following metrics: accuracy, recall, precision, sensitivity, specificity, area under curve, and F1-score. Euclidean distance was used to evaluate the resemblance between images, and we computed the contrastive loss function, which was then simplified to an

(14) $$L\left(W,Y,I_{1}I_{2}\right)=\left(1-Y\right)\;*\;\frac{1}{2}D^{2}+\left(Y\right)\;*\;\frac{1}{2}\;{\left[max(0,margin-D)\right]}^{2}$$

where *D* is the Euclidean distance between two similar or dissimilar images, and W represents the mutual parameter vector in neural networks. $I_{1}$ and $I_{2}$ represent the images. *Y* indicates whether the two images $(I_{1}$ and $I_{2})$ are similar (Y=0) or dissimilar (Y=1). Equation (15) expresses the Euclidean distance with respect to the images

(15) $$||f\left(I_{1}\right)\;-\;f\left(I_{2}\right){\left|\right|}^{2}$$

where $f(I_{1})$ and $f(I_{2})$ represent the latent representation of the input $I_{1}$ and $I_{2},$ respectively.

The COVID-19 identification network is constructed using super-resolution HR imagery. We trained this network on the NVIDIA GTX1080. Keras is used for the construction of the COVID-19 scheme. To construct our batch, we paired a single image with ten separate images. If the images were the same, we labeled the pair as 1, else, we labeled it 0. This pairing process was repeated for a total of 400 images and thus amounted to 4000 training pairs. This is one of the significant advantages of the siamese neural network. We can generate a large number of training pairs using a relatively smaller number of training images. In this work, we adopted a pre-trained VGG 16 model as the backbone for the siamese capsule network as seen in Figure 3. L1-norm distance utilized in this work calculates the difference between the two embeddings. In addition, finally, we used a dense layer with sigmoid activation to predict the output as 0 or 1 depending on whether the two images are similar or not. However, the remaining 298 images were used to test the performance of our proposed architecture.

## 4. Results

### 4.1. Experimental Setup

In investigating the performance of our proposed architecture on COVID-19 screening diagnosis, we sorted for an open domain dataset of CT images called the COVID-CT dataset. The authors in [39] obtained the dataset from 216 patients that consist of 349 COVID-19 CT images and 463 non-COVID-19 CTs. With the variation in sizes, the minimum to maximum height is between 153 and 1853, whereas the minimum to maximum width is between 124 and 1485. Furthermore, in avoiding over-fitting and enabling our dataset to train well, we balanced our dataset by selecting only 349 cases from each class.With the total of 698 images, 298 images were taken as a test set and 400 images for training, of which 400 images were paired with 10 distinct images, which amounted to 4000 images, hence 3000 images for the training set and 1000 images for the validation set.

### 4.2. Evaluation

This comprises of two parts—first, to illustrate the super-resolution network’s benefits in the image generation process. The second phase reports the performance metric of the proposed method. The evaluation criterion adopted as the metric to examine the diagnostic performance of our COVID-19 identification framework is as follows: accuracy (ACC), sensitivity (SEN), specificity (SPE), precision (PREC), F1-score, and the area under curve (AUC) represented from Equations (16)–(20):

(16) $$Accuracy=\frac{TP+TN}{TP+TN+FP+FN}$$

(17) $$Sensitivity=\frac{TP}{TP+FN}$$

(18) $$Specificity=\frac{TN}{TN+FP}$$

(19) $$Precision=\frac{TP}{TP+FP}$$

(20) $$F1-score=2*\frac{Precision*Recall}{Precision+Recall}$$

*TN*, *TP*, *FP*, and *FN* represent true negative, true positive, false positive, and false negative, respectively.

### 4.3. Super-Resolution Evaluation

Table 1 illustrates the efficacy of the super-resolution networks for the purpose of the reconstructed high-resolution task. The MESRGAN+ generates more suitable images, eliminates unimportant details and artifacts, and enhances extracting feature visibility. The HR images created by the MESRGAN+ preserve knowledge about the lung region while discriminating against distracting backgrounds.

**Figure 4:**
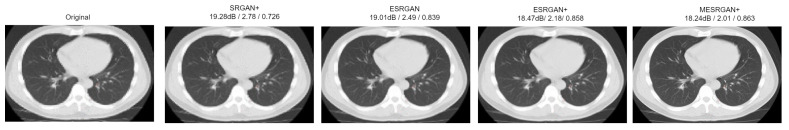
A quantitative comparison results of our proposed model, MESRGAN+, and other selected state-of-the-art models with the same dataset. The PSNR is reported on the left, Perceptual index value is reported in the middle, and the SSIM is reported on the right.

Furthermore, Figure 4 shows the performance of our proposed super-resolution, MESRGAN+, and other state-of-the-art models which are SRGAN, ESRGAN, and ESRGAN+. For fair comparison, we employed their available source code with our COVID-CT dataset. One of the aims of this research is to check the PSNR and perceptual index of the super-resolution models in which our model gives the best results in both cases. MESRGAN+ produces more appropriate images, removes artifacts, and improves extracting features clarity by extending the convolutional layer of the generative structure of the residual block and removing batch normalization.

### 4.4. COVID-19 Identification Evaluation

#### 4.4.1. Ablation Study

To start with, we performed an ablation study on the different network design of MESRGAN+ Siamese-CapsNet on the COVID-CT dataset. Particularly, we made a comparison about the following two architectures:

(1) CapsNet without max-pooling (replaced with DWT pooling): In this network, the max-pooling layers in VGG16 which serve as the feature extractor were totally replaced with DWT pooling, except for the first convolutional block in order to save the location information of the features.

(2) CapsNet with max-pooling: In contrast to the above network, the max-pooling layers were kept the same without making any changes to the VGG16 pre-training architecture.

The experimental results are summarized in Table 2. At first, we evaluated our proposed network using a CT dataset to examine the effect of pooling layers on the performance accuracy of the model. The pooling layers were removed one after the other leaving only the max-pooling layer in the first block. CapsNet with max-pooling layers replaced with DWT pooling had 2.28% higher accuracy than that with max-pooling layers. More to the point, the strategy of replacing the max-pooling layer with DWT pooling enhances the performance of the CapsNet by an accuracy of 2.28% in the COVID-CT dataset. It is evident that the CapsNet with max-pooling layers tends to lose more feature details; hence, it leads to a decrease in the network accuracy.

In addition, we examined the effect of regularization to network robustness by conducting another set of experiments on our proposed MESRGAN+ Siamese-CapsNet. Particularly, we made comparison on the following two architectures:

(1) MESRGAN+ Siamese-CapsNet without regularization: In this model, we remove the regularization.

(2) MESRGAN+ Siamese-CapsNet with regularization: Contrary to the model above, we keep the regularization.

The experimental results are summarized in Table 3. From all indications, the presence of regularization enhances the robustness of our proposed model.

#### 4.4.2. Results of the Proposed Model

**Figure 5:**
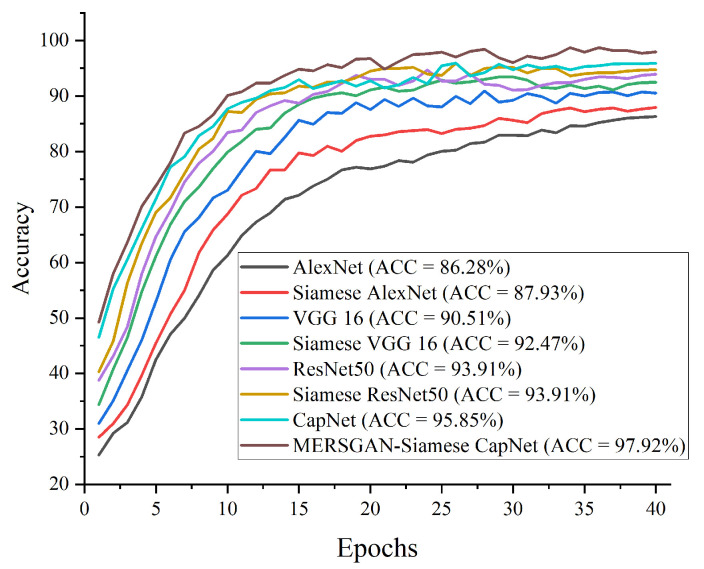
Performance accuracy in comparison with our proposed model and other pre-trained models for COVID-19 identification.

**Figure 6:**
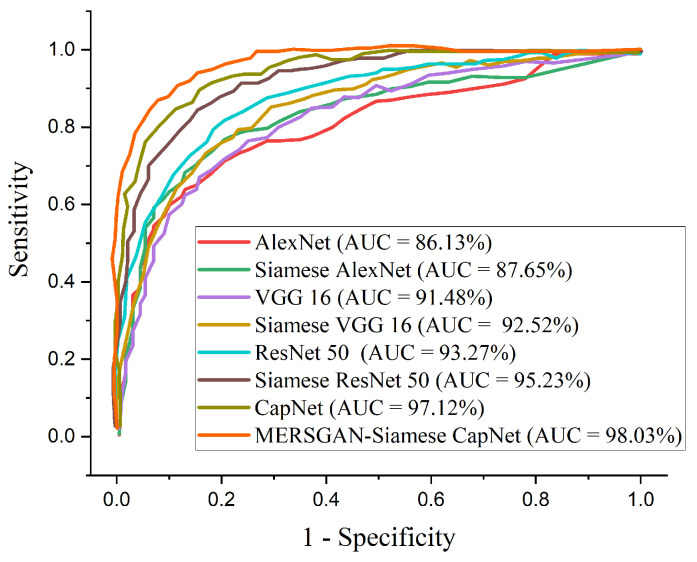
Performance ROC in comparison with our proposed model and other pre-trained models for COVID-19 identification.

**Figure 7:**
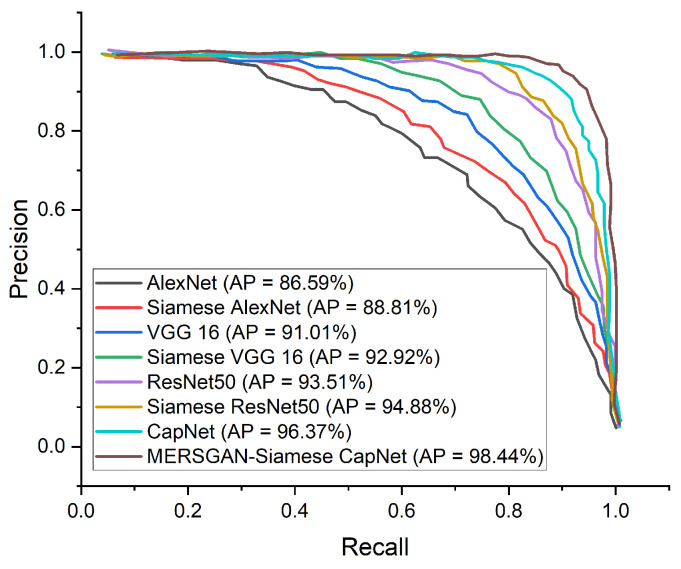
Performance precision–recall curve in comparison with our proposed model and other pre-trained models for COVID-19 identification.

In this section, we adopted ROC and precision–recall curves as the evaluation criteria to examine how well our model performs in comparison with a famous CNN model such as AlexNet, VGG16, and ResNet50. Table 4 presents the performance metrics on the COVID-CT dataset. Figure 5 reports the performance accuracy of our proposed model, showing it converges at an epoch of 15. Furthermore, Figure 6 presents the ROC curves for the models, and the precision–recall curves for the models are presented in Figure 7. Using an epoch of 40, our proposed MESRGAN+ Siamese-CapsNet obtains better identification performance compared to the selected pre-trained models. Our work achieves identification accuracy of 97.92%, sensitivity of 98.85%, specificity of 97.21%, AUC of 98.03% precision of 98.44%, and F1-score of 97.52%. Despite the close similarity in the lung areas which might pose some sort of difficulties as depicted in Figure 1, our proposed model still obtained high accuracy with less computational cost and robustness in strength. Additionally, the receiver operating characteristic curves provide a well-informed procedure for decision-making and offer better understanding to a radiologist in reducing the amount of false positives by balancing the specificity and sensitivity curves as presented in Figure 6. Furthermore, the precision–recall graph demonstrates the trade-off between precision and sensitivity. It is obvious that our model performs better than the other pre-trained models as shown in Figure 7, which means that our model has higher precision associated with higher sensitivity. The proposed model achieves the SOTA results by adopting a few parameters as compared to other SOTA algorithms. Our model algorithm is lightweight, cost-effective, and yet maintains the optimal task with a Siamese approach. These excellent outcomes depict how effective our architecture obtains accurate and robust screening of COVID-19.

#### 4.4.3. Compare Procedures

We compared the outcomes of our proposed algorithm with published State of the art (SOTA) COVID-19 screening methods which are as follows: Song et al. [26], Tang et al. [27], Wang et al. [12], Zheng et al. [18], Shi et al. [23], Jin et al. [24], and Xu et al. [28]. Table 5 presents SOTA results based on Chest radiographs for a COVID-19 diagnosing task. Song et al. [26] reported a deep learning diagnostic technique based on CT images called DeepPneumonia, where they used 88 CT data of confirmed COVID-19 patients from two different hospitals in China. This study reported an accuracy of 86%. Tang et al. [27] adopted a machine learning based method to automatically measure severity of COVID from CT exams belonging to 176 patients. This study reported 87.5% accuracy. Wang et al. [12] proposed a traditional residual CNN which aimed at differentiating COVID-19 from healthy and other Pneumonia CXR images and achieved an accuracy of 93.3%. Zheng et al. [18] reported a hybridized 3D classification CNN integrated U-Net segmentation algorithm which effectively detects the existence of COVID-19 with an accuracy of 90.1%. Shi et al. [23] proposed an infection region specific segmentation technique based on a random forest model to detect COVID-19 from other pneumonia using CT exams and the study reported 89.4% accuracy. Jin et al. [24] built and deployed an AI system which automatically analyze CT images to detect COVID-19 pneumonia characteristics using training samples of 1136 and achieved accuracy of 95.5%. Xu et al. [28] proposed an AI-based technique to screen coronavirus from healthy and viral pneumonia (Influenza-A) using CT exams. Nevertheless, the major flaws of their papers are that they neglected the fact that CT images are characterized with low quality that reduces the performance of an AI-based image diagnosis technique, which cannot really reflect the accuracy performance of COVID-19 diagnosis task. Furthermore, we selected a few recent state-of-the-art models and conducted a fair comparison using the same dataset as depicted in Table 6.

## 5. Discussion

COVID-19 has become a serious threat to health, and numerous solutions have been gathered together in combating it globally. Meanwhile, there are limited COVID-19 images in CT as compared to healthy images or other pneumonia—in as much as there is difficulty for humans to accurately differentiate COVID-19 CT images from other diseases or healthy images. The low-resolution and distortion of CT images have also contributed to the factors affecting the accuracy of detecting COVID-19 from other pneumonia. Therefore, we have presented an AI-based method for diagnosing COVID-19, which is an integration of modified enhanced super-resolution generative adversarial network plus (MESRGAN+) and the Siamese capsule network. First, the purpose of the modified enhanced super-resolution GAN plus is to achieve a higher-resolution from a low-resolution counterpart of the CT images. The HR output from MESRGAN+ module is fed into the Siamese capsule network in an end-to-end framework to learn the distinctive features for the identification of COVID-19.

Our proposed identification network is a lightweight Siamese capsule network with VGG16 pre-trained network as the backbone with shared parameters and weights for COVID-19 screening. The identification network determines the verification and identification loss simultaneously on the premise of a pair of CT training images. The network learns discriminative features and estimates the similarity score to determine whether the pair of input CT images includes the same scans or not. Additionally, a lot of papers have reported the possible causes of gradient-related bottlenecks and high computational cost due to architectural complexity. This paper utilizes a simple dual network of convolutional capsule network constructed using a VGG16 pre-trained model to extract high-level features which are fed to the primary capsule containing several convolutional capsule layers and finally connected to the fully connected layers used for aggregating the features extracted. L2 normalization term is utilized to normalize the CNN embeddings from which the contrastive loss function and Euclidean distances metric are used to estimate the distances and similarity scores between two CT scans.

It is well considered that the decline in performance of image-based COVID-19 diagnosis is because of the low quality dataset of CT. However, this argument is one-sided because it is evident that a deeper convolutional network suffers some setbacks such as exploding and vanishing problems. Therefore, we conclude that data quality and architectural complexity are equally responsible for the poor performance in the AI-based COVID-19 image screening task. To overcome these bottlenecks, we combined the modified enhanced super-resolution generative adversarial network plus (MESRGAN+) and the Siamese capsule network in an end-to-end framework for COVID-19 diagnosis. Based on the well-known evaluation metrics, the proposed COVID-19 identification network outperforms SOTA methods as illustrated in Table 5. Generally, our proposed algorithm consistently obtains better results using the standard evaluation metrics such as ACC, SEN, SPE, AUC, PREC, and F1-score.

## 6. Conclusions

In our paper, we proposed a joint framework of a modified enhanced super-resolution generative adversarial network plus (MESRGAN+) and the Siamese capsule network in an end-to-end framework for COVID-19 identification. First, the purpose of the modified enhanced super-resolution GAN plus is to achieve a higher-resolution from a low-resolution counterpart of the CT images. The HR output from MESRGAN+ module is fed into the Siamese capsule network in an end-to-end framework to learn the distinctive features for the identification of COVID-19. Furthermore, we explore the advantage of a capsule network and discrete wavelet transform pooling for obtaining spatial details of features and learn the distinctive features to deal with the discriminative problems of radiograph images by introducing Siamese learning technique in order to solve the problem of an insufficient CT dataset. In order to enhance the network robustness, we introduce a regularization term. From the experimental results, our proposed model is effective and converges very fast with better classification performance. In the future, we will focus on implementing one-shot learning in our model. To our knowledge, this is the first study to combine modified enhanced super resolution GAN plus with a Siamese capsule network as a cooperative learning method for a COVID-19 identification task from CT scans. We have demonstrated that our model can create more reasonable and real images, as well as capture deep features for COVID-19 identification. With a wide margin, our proposed approach outshone some of the SOTA COVID-19 screening techniques achieving an accuracy of 97.92%, sensitivity of 98.85%, specificity of 97.21%, AUC of 98.03%, precision of 98.44%, and F1 score of 97.52%, which is helpful for COVID-19 screening.

There are some drawbacks to this study; firstly, the perceptually compelling reconstruction of images is a difficult task that will be addressed in the future. The construction of content loss functions that characterize picture spatial content but are less sensitive to changes in pixel space may further enhance realistic image SR outcomes. Secondly, COVID-19 symptoms may resemble those of other pneumonia such as viral pneumonia, bacterial pneumonia, and so on. We solely compared CT exams of COVID-19 infection to that of healthy CT. The patient’s contact history, travel history, early symptoms, and laboratory evaluation are still required for a COVID-19 clinical diagnosis. Thirdly, the number of model samples in this investigation was limited. To improve accuracy in the future, the quantity of training and test samples should be increased. To deal with the complex clinical condition, more multi-center clinical trials should be done.

## Figures and Tables

**Table 1:** Comparison of the structural configuration of SRGAN, ESRGAN, ESRGAN+, and our proposed MESRGAN+ including their reported PSNR and Perceptual Index using a COVID-CT dataset.

Parameter	SRGAN	ESRGAN	ESRGAN+	MESRGAN+
Residual block of the generator	Conv(3, 64, 1) Batch norm ReLU Conv(3, 64, 1) Batch norm	Conv(3, 64, 1) ReLU Conv(3, 64, 1)	Conv(3, 64, 1) ReLU Conv(3, 64, 1)	Conv(3, 64, 1) ReLU Conv(1, 64, 1) ReLU Conv(3, 64, 1) ReLU Conv(3, 64, 1)
Input size	LR	LR	LR	LR
PSNR	19.28 dB	19.01 dB	18.47 dB	18.24 dB
Perceptual Index	2.78	2.49	2.18	2.01
SSIM	0.726	0.839	0.858	0.863

**Table 2:** Comparing the effect of DWT-pooling and Max-pooling on both the Capsule Network and Siamese Capsule Network in terms of performance accuracy.

Model	With DWT-Pooling	With Max-Pooling	Difference
	ACC (%)	ACC (%)	ACC (%)
Capsule Network	93.92	91.64	2.28
Siamese Capsule Network	97.10	94.89	2.21

**Table 3:** Comparing the effect of regularization on both the Capsule Network and Siamese Capsule Network in terms of performance accuracy.

Model	With Regularizer	W/o Regularizer	Difference
	ACC (%)	ACC (%)	ACC (%)
Capsule Network (Max-pooling)	92.79	91.64	1.15
Capsule Network (DWT-pooling)	94.66	93.92	0.74
Siamese Capsule Network (Max-pooling)	96.03	94.89	1.14
Siamese Capsule Network (DWT-pooling)	97.92	97.10	0.82

**Table 4:** Performance evaluation metrics for the proposed model in comparison with other methods using the same dataset.

Model	ACC (%)	SEN (%)	SPE (%)	AUC (%)	PREC (%)	F1-Score (%)
AlexNet	86.28	86.64	85.81	86.13	86.59	86.62
Siamese AlexNet	87.93	88.77	87.01	87.65	88.81	88.99
VGG 16	90.51	91.70	89.23	91.48	91.01	91.36
Siamese VGG 16	92.47	92.89	93.13	92.52	92.92	92.86
ResNet50	93.91	93.64	91.77	93.27	93.51	93.48
Siamese ResNet50	94.72	94.37	95.58	95.23	94.88	94.62
Capsule Network	95.85	96.41	95.94	97.12	96.37	96.49
MERSGAN-Siamese CapNet	97.92	98.85	97.21	98.03	98.44	97.52

**Table 5:** Performance comparison with other state-of-the-art models with our proposed model.

Model	ACC (%)	SEN (%)	SPE (%)
Song et al. [26]	86.0	96.0	77.0
Tang et al. [27]	87.5	93.3	74.5
Wang et al. [12]	93.3	91.4	90.5
Zheng et al. [18]	90.1	90.7	91.1
Shi et al. [23]	89.4	90.7	87.2
Jin et al. [24]	95.2	97.4	92.2
Xu et al. [28]	86.7	87.9	90.7
MERSGAN-Siamese CapNet	97.92	98.85	97.21

**Table 6:** Performance comparison of our proposed model with selected COVID-19 models using the same COVID-19 CT dataset.

Model	ACC (%)	SEN (%)	SPE (%)
Zheng et al. [18]	92.77	91.83	92.05
Shi et al. [23]	90.31	90.94	89.62
Jin et al. [24]	96.86	97.09	90.17
Xu et al. [28]	87.88	89.25	91.42
MERSGAN-Siamese CapNet	97.92	98.85	97.21

## Data Availability

In this study, we used an open-source dataset called the COVID-CT dataset. The dataset is gotten from Yang et al. [39] and consists of 349 COVID-19 CT images from 216 patients and 463 non-COVID-19 CTs. These CT scans have different sizes, whereby the minimum to maximum height are between 153 and 1853, whereas the minimum to maximum widths are between 124 and 1485.
